# Supplementary material for: Pilot Study to Detect Genes Involved in DNA Damage and Cancer in Humans: Potential Biomarkers of Exposure to E-Cigarette Aerosols
Source: Genes (Basel). 2021 Mar 22;12(3):448. doi: 10.3390/genes12030448 (PMC8004185; doi:10.3390/genes12030448)
Supplement: Supplementary file 1 [file genes-12-00448-s001.pdf]

# Supplementary Information

# Pilot study to detect genes involved in DNA damage and cancer in humans: Potential biomarkers of exposure to E-cigarette aerosols

Samera H. Hamad[1,2*], Marielle C. Brinkman[3], Yi-Hsuan Tsai[1], Namya Mellouk[4], Kandice Cross[5], Ilona Jaspers[2,6], Pamela I. Clark[7*], and Courtney A. Granville[8*]

[1]UNC Lineberger Comprehensive Cancer Center, University of North Carolina at Chapel Hill, Chapel Hill, NC27599
[2]Curriculum in Toxicology and Environmental Medicine, University of North Carolina at Chapel Hill, Chapel Hill, NC27599
[3]College of Public Health, Ohio State University, Columbus, OH43210
[4]National Institute of Environmental Health Sciences, Research Triangle Park, NC27709
[5]Gad Consulting Services, Risk Assessment, Consulting in Raleigh, NC27609
[6]Department of Pediatrics, School of Medicine, University of North Carolina at Chapel Hill, Chapel Hill, NC27514
[7]School of Public Health, University of Maryland, College park, MD20742
[8]Drug Information Association, Washington, DC20036

***Corresponding Authors: To whom correspondence should be addressed:**

**Samera Hamad**, UNC-Lineberger comprehensive Cancer Center, 450 West Dr. Chapel Hill, NC 27599, Tel: 608-217-2829, email: samera_hamad@med.unc.edu

**Pamela Clark**, School of Public Health, University of Maryland, College park, MD 20742, Tel: 443-791-3553, email: clarkp@umd.edu

**Courtney Granville**, Drug Information Association, Washington, DC 20036, Tel: 614-607-2766, email: courtneygranville@gmail.com

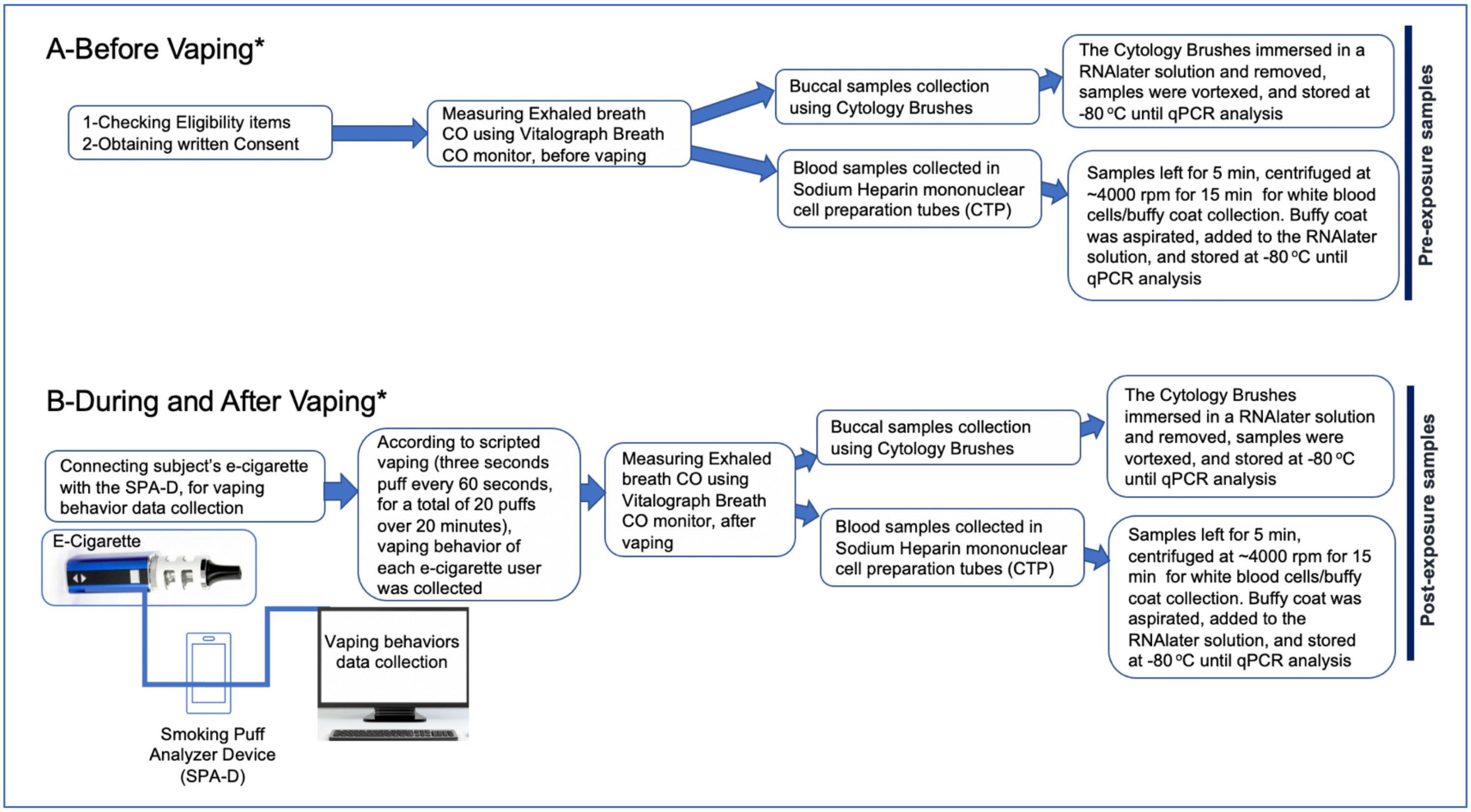


Supplementary Figure S1. The overview of the experimental study design, before vaping (A), and during and after vaping (B). [*] Blanks and duplicates of biological samples were collected in each session

Supplementary Table S1. Eligibility criteria

| Eligibility item | Eligible for Study Participation |
|---|---|
| Age | 18-59 years old |
| Type of E-cig | Cartomizer or tank style |
| Combustible cigarette smoker | No; should quit at least 2 months before participation |
| Past cigarette smoker | If yes, at least 100 cigarettes in lifetime; must have quit at least 2 months before participation in the study |
| Daily e-cig use | Yes |
| E-liquid-Levels of nicotine level | Maximum 6 mg/ml |

Supplementary Table S2. Eligibility screening survey; *DK: Do not Know

| **QUESTION** | **RESPONSE** | | | | **ELIGIBLE RESPONSE** |
|---|---|---|---|---|---|
| How did you find out about this study | Newspaper | Poster or Flyer | Friend | Other | |
| How old are you? | Age: | | Comments | | ≥18 ½ years |
| Do you use electronic cigarettes every day? | Yes | No | | | Yes |
| How long have you been using electronic cigarettes? | | | | | ≥2 months |
| How many times do you use an electronic cigarette per day | | | | | ≥8 |
| Are you willing to use your own electronic cigarette during a test session in our lab? | Yes | No | | | Yes |
| What type of electronic cigarette do you vape?<br><br>Brand, style, model, etc. | | | | | Will not determine eligibility |
| Do you use an electronic cigarette containing nicotine? | Yes | No | DK* | | Yes |
| Are you willing to use a commercially available electronic cigarette during a test session in our lab? | Yes | No | | | Yes |
| Could you come to the lab twice, one week apart, for about 3 hours each time? | Yes | No | | | Yes |
| Are you willing to use a recording device when you vape electronic cigarettes on your own time for one week? | Yes | No | | | Yes |
| Do you have any mouth or throat problems that would keep you from comfortably vaping an electronic cigarette? | Yes | No | | | No |
| Do you have a history of problems resulting from blood draws? | Yes | No | | | No |
| Will you have difficulty having two small samples (~2 teaspoons each) of your blood drawn during each lab visit? | Yes | No | | | No |
| Do you have respiratory allergies, a history of pulmonary disease, or asthma? | Yes | No | | | No |
| Do you suffer from claustrophobia, i.e., are you uncomfortable in closed-in spaces? | Yes | No | | | No |
| Are you in the process of trying to stop using electronic cigarettes? | Yes | No | | | No |
| WOMEN: Are you pregnant, breastfeeding, or trying to become pregnant? | Yes | No | | | No |

Supplementary Table S3. Laboratory visit dates and subjects' e-cigarettes vaped in this study

| Subject ID_ Male/Female | Visit # (date) | E-Cigarette Type (nicotine Conc. mg/ml) | CO Conc. ppm; before/ after vaping |
|---|---|---|---|
| S01_M | V1 (05/18/2017) | EVOD brand (6) | 0/0 |
| | V2 (05/23/2017) | | 0/0 |
| | V3 (05/25/2017) | | 0/0 |
| S02_F | V1 (05/22/2017) | Vaporfit Pro brand (3) | 1/1 |
| | V2 (06/05/2017) | | 1/0 |
| | V3(06/06/2017) | | 0/0 |
| S03_M | V1 (05/30/2017) | Smok Alein 220 brand (6) | 0/0 |
| | V2 (06/02/2017) | | 0/0 |
| | V3 (06/05/2017) | | 0/0 |

Supplementary Table S4. Number of buccal and blood samples, duplicates, and method blanks collected

| Subject ID_ Male/Female | Visit number | Sampling date | Number of Blood¥ samples | Number of Buccal¥ Samples | Duplicates (Dup) and Method Blanks (Blk)* |
|---|---|---|---|---|---|
| S01_M | V1 | 05/18/17 | 3 | 2 | Pst**-Blood Dup + One Buccal Blk |
| | V2 | 05/23/17 | 3 | 3 | Pre***-Blood Dup + Pst Buccal Dup |
| | V3 | 05/25/17 | 2 | 3 | Pre-Buccal Dup |
| S02_F | V1 | 05/22/17 | 3 | 3 | Pre-Blood Dup + Pst Buccal Dup |
| | V2 | 06/05/17 | 2 | 3 | Pre-Buccal Dup |
| | V3 | 06/06/17 | 3 | 2 | Pst-Blood Dup |
| S03_M | V1 | 06/02/17 | 3 | 2 | Pst-Blood Dup |
| | V2 | 06/05/17 | 2 | 2 | None |
| | V3 | 06/07/17 | 2 | 3 | Pre-Buccal Dup + One Buccal BLK + 2 Blood Blks |
| Total samples: | | | 23 + 2Blks=25 | 23 + 2Blks=25 | |

* Blanks of blood or buccal was performed by using RNA-Later solution only; **Pst: post exposure; ***Pre: pre-exposure

## SUPPORTING MATERIALS AND METHODS

***Quantitative RT-PCR analysis of gene expression*:** The fifty biologic (25 buccal epithelial and 25 blood) samples were shipped to Battelle Eastern Science and Technology (BEST), June 26, 2017, and extracted for RNA. The RNA extraction was performed using the RNeasy Mini Kit, followed by cDNA synthesis using the $RT^2$ First Strand Kit (Qiagen, Alameda, CA, USA). After cDNA synthesis, samples were diluted in water and aliquoted within the 96-well array. The Human DNA Damage Signaling Pathway RT2 Profiler TM PCR Array (QIAGEN, PAHS-029ZA) comes pre-loaded with SYBR-based primers in each well that are specific to 84 DNA damage related genes , 5 house-keeping genes [Beta-2-Microglobulin (B2M), Hypoxanthine phosphoribosyl transferase 1 (HPRT1), Ribosomal Protein L13a (RPL13A), Glyceraldehyde-3-phosphate Dehydrogenase (GAPDH), and Actin beta (ACTB), a human genomic DNA contamination negative control (HGDC), reverse transcription control (RTC) and positive PCR controls (PPC). Positive control RTC and PPC genes had robust expression with approximate cycle threshold (Ct) values in the 18 to 26 range for all samples.

The fold changes for each of the 84 genes relative to each of the five house-keeping genes was summarized in the accompanying Excel files. For buccal (BUCL) samples the house-keeping gene B2M had relatively robust signaling according to Ct values (average of 26); for WBC/blood samples, while HPRT1 was the most appropriate house-keeping gene for the respective 25 buccal samples. The reportable values for each sample from each array was expressed as a relative cycle threshold (Ct) value and converted to fold-change values relative to appropriate house-keeping genes.

Supplementary Table S5. Genes, their abbreviations, and the catalog numbers of their primers, examined in the PCR-bioassay

| GENE ID | GENE OFFICIAL NAME | RT2 qPCR ASSAY CATALOG # |
|---|---|---|
| ABL1 | Tyrosine-protein kinase ABL1 | PPH00087E |
| ANKRD17 | Ankyrin repeat domain-containing protein 17 | PPH12069A |
| APEX1 | DNA-apurinic or apyrimidinic site lyase | PPH02201A |
| ATM | Ataxia Telangiectasia Muted | PPH00325C |
| ATR | Ataxia Telangiectasia And Rad3 Related | PPH01318B |
| ATRX | Alpha thalassemia/metal retardation syndrome X-linked | PPH00470F |
| BRCA1 | Breast cancer type 1 susceptibility protein | PPH00322F |
| BTG2 | BTG anti-proliferation factor 2 | PPH01750C |
| CCNH | Cyclin-Dependent Kinase-Activating Kinase Complex Subunit | PPH00959E |
| CDK7 | Cyclin Dependent Kinase 7 | PPH00935E |
| CHEK1 | Checkpoint Kinase 1 | PPH00940C |
| CHEK2 | Checkpoint Kinase 2 | PPH00921B |
| CIB1 | Calcium And Integrin Binding 1 | PPH12320C |
| CIDEA | Cell Death Inducing DFFA Like Effector A | PPH00899C |
| CRY1 | Cryptochrome Circadian Regulator 1 | PPH06231B |
| DDB1 | Damage Specific DNA Binding Protein 1 | PPH01515A |
| DDIT3 | DNA Damage Inducible Transcript 3 | PPH00310A |
| DMC1 | DNA Meiotic Recombinase 1 | PPH02712F |
| ERCC1 | Excision Repair 1, Endonuclease Non-Catalytic Subunit | PPH01539A |
| ERCC2 | ERCC Excision Repair 2, TFIIH Core Complex Helicase Subunit | PPH01550C |
| EXO1 | Exonuclease 1 | PPH02715A |
| FANCG | FA Complementation Group G | PPH20387A |
| FEN1 | Flap Structure-Specific Endonuclease 1 | PPH00502B |
| XRCC6 | X-Ray Repair Cross Complementing 6 | PPH02175A |
| GADD45A | Growth Arrest And DNA Damage Inducible Alpha | PPH00148B |
| GADD45G | Growth Arrest And DNA Damage Inducible Gamma | PPH02207A |
| GML | Glycosylphosphatidylinositol Anchored Molecule Like | PPH01755F |
| GTF2H1 | General Transcription Factor IIH Subunit 1 | PPH02600F |
| GTF2H2 | General Transcription Factor IIH Subunit 2 | PPH73147A |
| GTSE1 | G2 And S-Phase Expressed 1 | PPH01748F |
| HUS1 | HUS1 Checkpoint Clamp Component | PPH00922B |
| IGHMBP2 | Immunoglobulin Mu DNA Binding Protein 2 | PPH08993F |
| IP6K3 | Inositol Hexa-kis-phosphate Kinase 3 | PPH08133A |
| XRCC6BP1 | ATP23 Metallopeptidase And ATP Synthase Assembly Factor Homolog | PPH09665E |
| LIG1 | DNA Ligase 1 | PPH02094A |
| MAP2K6 | Mitogen-Activated Protein 2 Kinase 6 | PPH00742B |

| | | |
|---|---|---|
| MAPK12 | Mitogen-Activated Protein Kinase 12 | PPH01779A |
| MBD4 | Methyl-CpG Binding Domain 4, DNA Glycosylase | PPH02709A |
| MLH1 | MutL Homolog 1 | PPH00196F |
| MLH3 | MutL Homolog 3 | PPH02700A |
| MNAT1 | MNAT1 Component Of CDK Activating Kinase | PPH02714A |
| MPG | N-Methylpurine DNA Glycosylase | PPH02102A |
| MRE11A | MRE11 Homolog, Double Strand Break Repair Nuclease | PPH01097C |
| MSH2 | MutS Homolog 2 | PPH00197E |
| MSH3 | MutS Homolog 3 | PPH02195A |
| MUTYH | MutY DNA Glycosylase | PPH02697E |
| N4BP2 | NEDD4 Binding Protein 2 | PPH19003B |
| NBN | Nibrin | PPH00946C |
| NTHL1 | Nth Like DNA Glycosylase 1 | PPH02720A |
| OGG1 | 8-Oxoguanine DNA Glycosylase | PPH02103A |
| PCBP4 | Poly (RC) Binding Protein 4 | PPH01756C |
| PCNA | Proliferating Cell Nuclear Antigen | PPH00216B |
| AIFM1 | Apoptosis Inducing Factor Mitochondria Associated 1 | PPH01037A |
| PMS1 | PMS1 Homolog 1, Mismatch Repair System Component | PPH02155B |
| PMS2 | PMS1 Homolog 2, Mismatch Repair System Component | PPH02095E |
| PMS2P3 | PMS1 Homolog 2, Mismatch Repair System Component Pseudogene 3 | PPH02741C |
| PNKP | Polynucleotide Kinase 3'-Phosphatase | PPH02725A |
| PPP1R15A | Protein Phosphatase 1 Regulatory Subunit 15A | PPH02081E |
| PRKDC | Protein Kinase, DNA-Activated, Catalytic Subunit | PPH01309C |
| RAD1 | RAD1 Checkpoint DNA Exonuclease | PPH00741F |
| RAD17 | RAD17 Checkpoint Clamp Loader Component | PPH00929F |
| RAD18 | RAD18 E3 Ubiquitin Protein Ligase | PPH02740A |
| RAD21 | RAD21 Cohesin Complex Component | PPH10216A |
| RAD50 | RAD50 Double Strand Break Repair Protein | PPH00956B |
| RAD51 | RAD51 Recombinase | PPH00942F |
| RAD51B | RAD51 Paralog B | PPH02654B |
| RAD9A | RAD9 Checkpoint Clamp Component A | PPH00944C |
| RBBP8 | RB Binding Protein 8, Endonuclease | PPH00954F |
| REV1 | REV1 DNA Directed Polymerase | PPH21080A |
| RPA1 | Replication Protein A1 | PPH02730A |
| SEMA4A | Semaphorin 4A | PPH19432A |
| SESN1 | Sestrin 1 | PPH01759B |
| SMC1A | Structural Maintenance Of Chromosomes 1A | PPH14489A |
| SUMO1 | Small Ubiquitin Like Modifier 1 | PPH00973F |
| TP53 | Tumor Protein P53 | PPH00213F |
| TP73 | Tumor Protein P73 | PPH00725A |
| TREX1 | Three Prime Repair Exonuclease 1 | PPH02340B |
| UNG | Uracil DNA Glycosylase | PPH01727E |
| XPA | DNA Damage Recognition And Repair Factor | PPH01524C |

| | | |
|---|---|---|
| XPC | Complex Subunit, DNA Damage Recognition And Repair Factor | PPH01536F |
| XRCC1 | X-Ray Repair Cross Complementing 1 | PPH01741A |
| XRCC2 | X-Ray Repair Cross Complementing 2 | PPH01694A |
| XRCC3 | X-Ray Repair Cross Complementing 3 | PPH02208A |
| ZAK | Sterile alpha motif and leucine zipper containing kinase | PPH05541E |

Supplementary Table S6A. The expression data of eighty-four genes analyzed in human buccal samples

| GENE ID_Buccal | S01_V1 | S01_V2 | S01_V3 | S02_V1 | S02_V2 | S02_V3 | S03_V1 | S03_V2 | S03_V3 |
|---|---|---|---|---|---|---|---|---|---|
| ABL1 | 0.120309009 | 1.575353818 | 1.890530097 | 0.568191141 | 3.176353404 | 2231.628876 | 3.238911294 | 13.81549776 | 0.402849938 |
| ANKRD17 | 0.13200939 | 0.621804617 | 3.681536873 | 0.523439044 | 8.511715308 | 10.61202304 | 2.061779428 | 0.007006468 | 0.001262416 |
| APEX1 | 331.0232118 | 3.435630297 | 2.38827987 | 0.457142576 | 0.689381733 | 6.384056903 | 2.061779428 | 2.815950212 | 1.266454738 |
| ATM | 0.098133556 | 0.507455341 | 9.433798232 | 0.549357985 | 4.826227769 | 47.14027618 | 7.20691785 | 1.619864637 | 0.599583071 |
| ATR | 0.146976155 | 2.081903632 | 10.95905862 | 0.715768408 | 4.0693953 | 5.732655374 | 1.883156232 | 1.952197933 | 0.946047691 |
| ATRX | 0.13200939 | 0.047860167 | 2.38827987 | 0.327209944 | 6.054709583 | 0.00606395 | 2.061779428 | 2.815950212 | 0.044373325 |
| BRCA1 | 0.033486775 | 0.987445738 | 2.38827987 | 2.044983543 | 0.146308947 | 18981.96874 | 0.001391494 | 2.815950212 | 0.023869228 |
| BTG2 | 0.318069937 | 77.57168264 | 3.160996439 | 0.651070681 | 0.230692978 | 1.886206514 | 0.832266203 | 56.36078783 | 2.395299775 |
| CCNH | 0.043200211 | 3.523177999 | 1.877575208 | 3.535515036 | 7.868484742 | 0.401584744 | 5.419608179 | 16.21225032 | 3.416940387 |
| CDK7 | 0.049782138 | 13.29625733 | 3.518151058 | 1.19699613 | 18.90785493 | 93.4142678 | 0.639300698 | 3.374159202 | 1.469479753 |
| CHEK1 | 0.771947945 | 3.520370405 | 2.775243364 | 0.009119023 | 3.0538533 | 0.01613128 | 3.630706911 | 0.203360883 | 0.817502628 |
| CHEK2 | 0.001682382 | 4.967004392 | 0.151624817 | 13.26195302 | 45.30274289 | 0.759034323 | 46.75066736 | 3.995803298 | 4.438646397 |
| CIB1 | 0.017293089 | 2.939631777 | 12.38991151 | 0.044443485 | 23.29191785 | 9.6456544 | 4.081750944 | 2.654260439 | 11.33955471 |
| CIDEA | 0.13200939 | 4.950176131 | 2.38827987 | 0.914501544 | 6.054709583 | 315.3146601 | 2.061779428 | 2.815950212 | 1.3246043 |
| CRY1 | 0.13200939 | 0.036410565 | 0.032957525 | 1120.480546 | 1.733152677 | 4.780552819 | 2.290474158 | 1.021078757 | 1.32930942 |
| DDB1 | 0.178217997 | 3.767706937 | 0.857658256 | 1.523887726 | 0.921712049 | 1.257812879 | 4.563563327 | 3.021071541 | 0.82171559 |
| DDIT3 | 0.13200939 | 3.435630297 | 2.38827987 | 0.327209944 | 6.054709583 | 6.384056903 | 2.061779428 | 2.815950212 | 0.039505301 |
| DMC1 | 0.010672731 | 3.435630297 | 0.073413402 | 0.468522087 | 6.054709583 | 3.302305315 | 2.061779428 | 2.815950212 | 0.191353877 |
| ERCC1 | 0.351517569 | 0.060570279 | 0.147928056 | 5.607316134 | 0.039094002 | 1.40012115 | 0.009576426 | 1.860525278 | 1.265987681 |
| ERCC2 | 0.068194589 | 0.083109084 | 14.03347657 | 8.987923365 | 3.694082346 | 8.756778057 | 58.91284543 | 0.330856563 | 5.814433026 |
| EXO1 | 0.39838033 | 1.794177661 | 2.518960539 | 0.12915611 | 12.69419838 | 48.42035898 | 3.287896747 | 1.753760216 | 1.998913551 |
| FANCG | 0.037200278 | 1.132324103 | 0.977885325 | 18.19844569 | 1.958324343 | 8.692042086 | 0.511956755 | 0.218433696 | 0.648344343 |
| FEN1 | 2.252745388 | 110.2662338 | 0.210669265 | 0.920755422 | 6.054709583 | 7.465073967 | 2.061779428 | 11.18701478 | 24.35435232 |
| XRCC6 | 0.13200939 | 6.026530466 | 0.037052095 | 542.8485328 | 0.011580974 | 0.002854539 | 0.001317913 | 1.800327 | 0.001532143 |
| GADD45A | 0.110732124 | 0.973303937 | 3.42341097 | 0.5354124 | 0.618465519 | 2.702835128 | 7.885177016 | 0.159381005 | 4.60229201 |
| GADD45G | 0.13200939 | 3.435630297 | 2.38827987 | 1.985517814 | 6.054709583 | 6.384056903 | 2.061779428 | 2.815950212 | 1.3246043 |
| GML | 0.13200939 | 3.435630297 | 2.38827987 | 0.327209944 | 6.054709583 | 6.384056903 | 2.061779428 | 2.815950212 | 1.3246043 |
| GTF2H1 | 0.13329841 | 1.711886535 | 3.795569599 | 0.686519529 | 7.458819049 | 16.47050409 | 2.206377674 | 2.354891439 | 2.563512392 |
| GTF2H2 | 0.377422815 | 166.2985917 | 1.58280675 | 0.327209944 | 10.17867543 | 5.078750181 | 6.899707788 | 2.815950212 | 1.3246043 |
| GTSE1 | 0.129233656 | 5.086139276 | 3.86190886 | 0.378737919 | 3.062969805 | 22.87107739 | 0.412263783 | 2.018103951 | 0.994394315 |
| HUS1 | 0.13200939 | 3.435630297 | 2.38827987 | 1.461999801 | 6.054709583 | 0.004874512 | 2.061779428 | 2.815950212 | 1.3246043 |
| IGHMBP2 | 0.101131041 | 0.012666843 | 24.42693852 | 2.210500315 | 2.122186025 | 1.160793805 | 0.024468767 | 1.010750303 | 1.350447306 |
| IP6K3 | 0.13657792 | 9.026338798 | 5.367788583 | 199.9484262 | 192.4642434 | 0.042944689 | 14.32814819 | 1.578153413 | 82.62898551 |
| XRCC6BP1 | 0.051863987 | 1.956864695 | 4.450975004 | 0.161331832 | 0.890986977 | 6.668257774 | 0.027761354 | 184.7309282 | 0.039510055 |
| LIG1 | 0.248132311 | 4.400811869 | 0.710651226 | 0.389600152 | 7.930345916 | 0.017407963 | 104.8214445 | 0.014001085 | 1.250715716 |
| MAP2K6 | 0.023650255 | 32.18018042 | 1.086010181 | 0.391385894 | 41.81235504 | 2.706038801 | 3.044873664 | 9.566959264 | 13.83381152 |
| MAPK12 | 0.138333374 | 3.247817393 | 1.921780876 | 0.757400733 | 7.04343841 | 38.92061127 | 4.687141234 | 4.273541942 | 1.57565376 |
| MBD4 | 0.13200939 | 0.001412489 | 2.38827987 | 0.327209944 | 6.054709583 | 6.384056903 | 2.061779428 | 8543.275421 | 0.00100669 |
| MLH1 | 0.017093063 | 22.70496989 | 3.290349279 | 0.000954389 | 3.502991571 | 13.091698 | 0.16134783 | 5.16790416 | 4.465681111 |
| MLH3 | 0.539246353 | 10.40046609 | 1.503824695 | 0.746779601 | 2.992343909 | 58.59670556 | 5.171341974 | 0.597745261 | 1.805699892 |
| MNAT1 | 0.11701877 | 4.340056813 | 0.056413725 | 1.719830776 | 6843.006653 | 165.2152342 | 0.848004655 | 10.17600447 | 1.3246043 |
| MPG | 0.050214571 | 6.760462929 | 18.66403418 | 0.154273703 | 0.469225853 | 17.84536995 | 0.018739671 | 0.298385436 | 20.71333601 |
| MRE11A | 0.453968584 | 1.734566002 | 2.540502272 | 0.273114674 | 5.478122727 | 1.824733597 | 2.295324366 | 16.1421424 | 1.243862457 |
| MSH2 | 0.028924873 | 3.435630297 | 0.057477033 | 3.098583751 | 6.054709583 | 44.81995911 | 2.061779428 | 2.815950212 | 0.633953614 |
| MSH3 | 0.000408053 | 116.5218076 | 2.616050039 | 0.087397282 | 1.860378849 | 0.00602301 | 0.375523003 | 0.205750986 | 0.551102396 |
| MUTYH | 0.070364982 | 2.079218993 | 2.201112959 | 0.387845119 | 20.34146042 | 125.4343017 | 2.688059682 | 1.639709159 | 2.067538945 |
| N4BP2 | 0.269595374 | 4.096293033 | 1.584050234 | 0.515117862 | 15.86827301 | 3.368172586 | 1.729782067 | 0.590968737 | 1.064826318 |
| NBN | 0.13176737 | 2.852136441 | 0.81897167 | 1.619198743 | 0.904679245 | 1.116038794 | 2.22035918 | 1.222906175 | 0.63881658 |
| NTHL1 | 0.102785223 | 3.435630297 | 2.38827987 | 15.58309882 | 3.743954664 | 6.384056903 | 2.061779428 | 2.815950212 | 1.3246043 |
| OGG1 | 0.019365535 | 31.30387497 | 0.711258424 | 0.109757489 | 17.69910421 | 0.16490203 | 32.07984692 | 40.04206207 | 2.307275228 |
| PCBP4 | 0.131828008 | 62.72406981 | 2.68502289 | 14.28594892 | 0.335132135 | 50.55740766 | 2.172988297 | 0.225245662 | 1.621885406 |
| PCNA | 0.13200939 | 3.435630297 | 2.38827987 | 0.327209944 | 6.054709583 | 6.384056903 | 2.061779428 | 2.815950212 | 1.3246043 |
| AIFM1 | 0.529027003 | 2.690331528 | 19.14684938 | 21.58209028 | 27.94763896 | 2.223229136 | 1.020777764 | 0.88595069 | 2.688919842 |
| PMS1 | 0.051538083 | 9.73198379 | 7.564050187 | 0.099268546 | 11.27608445 | 1.404625931 | 1.56648146 | 4.461662325 | 2.386007566 |
| PMS2 | 0.057670751 | 0.065785419 | 0.79486894 | 46.39459571 | 2.382810426 | 1.513228403 | 2.127646089 | 0.21538851 | 1.775238014 |
| PMS2P3 | 0.843408215 | 9.267125847 | 5.883820191 | 1.0286454 | 2.991975888 | 0.719458663 | 20.46930975 | 1.009277453 | 1.256213375 |
| PNKP | 0.13200939 | 0.008194754 | 2.38827987 | 0.327209944 | 8.597999703 | 6.384056903 | 2.6001784 | 757.1913712 | 0.731926332 |
| PPP1R15A | 0.128295319 | 1.365349454 | 6.352355448 | 0.869228687 | 9.11481922 | 0.891851583 | 1.646144994 | 0.893370093 | 0.768630073 |
| PRKDC | 0.009980137 | 0.828142562 | 1.729416202 | 0.061695861 | 6.054709583 | 6.384056903 | 0.774748358 | 24.92811421 | 1.3246043 |
| RAD1 | 0.120085265 | 4.493017108 | 2.438452584 | 0.266685655 | 7.927792849 | 6.792302425 | 2.005183946 | 2.929408907 | 1.671348484 |
| RAD17 | 0.011071764 | 0.121310765 | 0.02091673 | 102.4318243 | 0.005740002 | 0.314960936 | 0.006338674 | 0.961070847 | 0.524404269 |
| RAD18 | 0.13200939 | 3.435630297 | 2.38827987 | 0.327209944 | 6.054709583 | 6.384056903 | 3.349505179 | 2.815950212 | 1.3246043 |
| RAD21 | 0.13200939 | 3.435630297 | 2.38827987 | 0.327209944 | 6.054709583 | 0.011129044 | 2.061779428 | 2.815950212 | 0.036012637 |
| RAD50 | 0.13200939 | 3.435630297 | 1.72340198 | 0.327209944 | 6.054709583 | 6.384056903 | 2.061779428 | 0.882068191 | 1.3246043 |
| RAD51 | 0.13200939 | 3.435630297 | 0.081742053 | 5.873863928 | 2450.083489 | 0.004342792 | 0.484494974 | 3.709272271 | 0.785971957 |
| RAD51B | 0.063806792 | 5.419171124 | 1.847517068 | 7.227441906 | 4.806317192 | 43.2666999 | 11.89339131 | 0.436652589 | 2.210415566 |
| RAD9A | 0.495519855 | 3.511896037 | 1.27834786 | 578.7459662 | 4.83042156 | 0.009306623 | 213.288432 | 1.302661367 | 3.991167739 |
| RBBP8 | 0.13200939 | 3.435630297 | 2.38827987 | 0.327209944 | 6.054709583 | 2914.749076 | 2.061779428 | 2.815950212 | 0.05075701 |
| REV1 | 332.9517849 | 3.435630297 | 18.4199774 | 10.53006483 | 6.054709583 | 6.384056903 | 0.002026414 | 2.815950212 | 1.3246043 |
| RPA1 | 0.087548199 | 5.275393244 | 0.508836574 | 3.110589372 | 12.40014056 | 2.12566414 | 1.504114996 | 0.002236031 | 0.022672397 |
| SEMA4A | 0.083010144 | 3.435630297 | 1.38979915 | 0.327209944 | 0.111418273 | 6.384056903 | 0.12822054 | 3.25324936 | 0.054193794 |
| SESN1 | 0.060721014 | 1.230943576 | 1.261907027 | 1.718619298 | 17.97812655 | 1.702502109 | 2.659132063 | 1.473487293 | 8.854881074 |
| SMC1A | 0.005292859 | 1.565626369 | 0.329971106 | 0.932952568 | 0.439427787 | 1.117522181 | 2.534698308 | 1.611116434 | 0.839001947 |
| SUMO1 | 0.035529322 | 10.3565037 | 2.760288293 | 1.726756862 | 6.054709583 | 6.384056903 | 2.061779428 | 2.815950212 | 0.02896762 |
| TP53 | 0.578940431 | 2.983353253 | 0.115444936 | 3.587107848 | 115.8479575 | 21.5398561 | 2348.169666 | 6.979056065 | 45.51349845 |
| TP73 | 0.13200939 | 3.435630297 | 0.347283082 | 38.11605664 | 2.423442348 | 6.384056903 | 0.008500162 | 0.417063358 | 4.34361001 |
| TREX1 | 0.967948993 | 3.638505773 | 2.507344848 | 4.967496922 | 30.49202199 | 1.914781104 | 1.35715735 | 0.747445336 | 14.10719619 |
| UNG | 0.263795482 | 23.69860937 | 0.102467733 | 0.327209944 | 6.019141624 | 6.384056903 | 2.061779428 | 2.815950212 | 6.221524198 |
| XPA | 0.038500719 | 3.435630297 | 2.38827987 | 4.013311582 | 21.70373989 | 1.585262319 | 2.061779428 | 1.1692644 | 2.366407729 |
| XPC | 0.03008282 | 11.40614783 | 1.541201892 | 0.285839531 | 1.440598794 | 108.813424 | 9.542364844 | 4.029127694 | 1.455983589 |
| XRCC1 | 0.080607921 | 3.059323323 | 2.939433577 | 0.200712286 | 15.87626316 | 11.98660807 | 8.571356509 | 2.196740618 | 0.292607252 |
| XRCC2 | 1.26658032 | 3.435630297 | 2.38827987 | 0.792750053 | 1.016652725 | 10.14047383 | 16.31608517 | 3.504359287 | 1.3246043 |
| XRCC3 | 0.082172215 | 1.42429331 | 3.055640865 | 0.693654006 | 11.11530201 | 15.03783237 | 0.382627313 | 3.159964377 | 2.838034937 |
| ZAK | 0.092636356 | 3.941061573 | 5.233476787 | 0.378277537 | 4.088713384 | 2.676195597 | 1.610828908 | 0.730180823 | 2.000158655 |

Supplementary Table S6B. The expression data of eighty-four genes analyzed in human blood/White Blood Cells (WBC) samples

| GENE ID_WBC | S01_V1 | S01_V2 | S01_V3 | S02_V1 | S02_V2 | S02_V3 | S03_V1 | S03_V2 | S03_V3 |
|---|---|---|---|---|---|---|---|---|---|
| ABL1 | 2.2524312 | 0.931575842 | 0.934189286 | 0.898421717 | 0.470798394 | 0.411541689 | 0.819092946 | 337.2539959 | 2532.550819 |
| ANKRD17 | 0.066385602 | 0.835737219 | 0.767729245 | 0.581029912 | 0.740894922 | 0.010442532 | 0.170616229 | 0.627245735 | 2.191116485 |
| APEX1 | 0.007567257 | 39.84859063 | 2.304894106 | 0.729324575 | 0.298198507 | 0.002658644 | 112.785526 | 1.236371488 | 7.228387934 |
| ATM | 13.80780117 | 0.530592151 | 6.696840848 | 0.631369065 | 1.373091701 | 1.783679036 | 0.584596646 | 1806.745552 | 10235.9389 |
| ATR | 3.107410036 | 1.174435085 | 1.255885409 | 1.863996761 | 1.187723866 | 3.640690337 | 0.115985581 | 1486.314053 | 32273.01394 |
| ATRX | 0.12141257 | 0.903343051 | 0.894670249 | 0.9092447 | 0.871418338 | 0.038415961 | 0.264778187 | 0.319232247 | 114160.724 |
| BRCA1 | 0.014056754 | 29.96656926 | 0.652659865 | 0.920288095 | 3.429893807 | 0.009313075 | 38.48855017 | 2.044234778 | 87421.06196 |
| BTG2 | 2.915520817 | 0.734445122 | 1.060524413 | 1.158553845 | 0.786372117 | 0.176577218 | 0.090971007 | 47.37623141 | 7372.180072 |
| CCNH | 8.157810698 | 0.981868239 | 2.492933486 | 0.597577748 | 1.080433601 | 1.151530199 | 2.719044901 | 13.80423279 | 1035.770765 |
| CDK7 | 6.155983011 | 0.453809264 | 1.133099822 | 0.184842357 | 1.999555833 | 4.515807469 | 0.135978316 | 2627.995214 | 138.4197028 |
| CHEK1 | 1860.799287 | 726.6181135 | 0.055988201 | 0.023683078 | 0.044745995 | 0.100604425 | 0.364661564 | 174.278351 | 94948.94999 |
| CHEK2 | 0.239756044 | 18.75001503 | 0.000865349 | 5.495126797 | 0.001641937 | 0.049710976 | 0.034744843 | 27831.97746 | 3181.115081 |
| CIB1 | 140.0901616 | 0.264396201 | 1.755388628 | 5.205109771 | 1.086771414 | 4.887061069 | 0.004207051 | 13784.51638 | 498.7455333 |
| CIDEA | 6.308071033 | 0.244680375 | 0.652659865 | 2.618354488 | 1.108693766 | 0.161577091 | 0.025381961 | 900.4704613 | 11360.08028 |
| CRY1 | 0.158398016 | 0.536087056 | 0.250348609 | 0.992625697 | 1.776879322 | 0.075139167 | 60.61673372 | 0.629880854 | 17.07237292 |
| DDB1 | 2.418479491 | 0.421171451 | 0.657455074 | 1.267398583 | 0.454357667 | 1.46527388 | 0.272937442 | 229.2400663 | 1634.095236 |
| DDIT3 | 6.308071033 | 0.209165496 | 16.89545959 | 0.032511944 | 1.108693766 | 1.68161274 | 0.025381961 | 900.4704613 | 11360.08028 |
| DMC1 | 0.010589098 | 0.730946265 | 0.856970262 | 1.181140812 | 0.002664792 | 0.013465093 | 40.71510501 | 4.990621841 | 11360.08028 |
| ERCC1 | 0.616229401 | 0.903200345 | 1.038834601 | 0.481565081 | 1.478703664 | 0.469388837 | 0.902918582 | 0.565226442 | 16.90888948 |
| ERCC2 | 0.358647971 | 5.023896968 | 0.302232609 | 2.344249259 | 0.820123433 | 0.778461001 | 0.658290909 | 463.554769 | 72281.95392 |
| EXO1 | 0.396841956 | 1.204977935 | 0.984924173 | 0.772485974 | 1.367978391 | 1.332834246 | 0.03176808 | 352.7633422 | 21988.1771 |
| FANCG | 0.26824276 | 0.798587198 | 0.830187005 | 0.551952596 | 2.704267533 | 1.536479913 | 0.749154859 | 20.54191004 | 20289.35582 |
| FEN1 | 0.005490338 | 41.82831138 | 1.253380747 | 1.171575413 | 1.251986278 | 0.107206715 | 10.81815706 | 0.454834268 | 5187473.284 |
| XRCC6 | 219.2641975 | 0.122197775 | 1.184005048 | 0.652020795 | 0.432177461 | 0.012147624 | 31.95620063 | 1.065591016 | 3.753576972 |
| GADD45A | 1.359593449 | 3.230544234 | 2.175566772 | 0.611512507 | 0.011561525 | 0.173385075 | 22.06981466 | 104.1971989 | 2935.7902 |
| GADD45G | 0.006885015 | 32.19615987 | 1.163235709 | 0.32683337 | 1.108693766 | 0.013533232 | 46.37325532 | 1.634676726 | 11360.08028 |
| GML | 0.063156153 | 0.244680375 | 0.652659865 | 0.137895505 | 1.108693766 | 0.096422717 | 0.66687631 | 900.4704613 | 11360.08028 |
| GTF2H1 | 40.88255854 | 0.620493564 | 1.456543848 | 2.709685867 | 1.477173727 | 40.16724525 | 0.049917111 | 3420.799962 | 7498.513136 |
| GTF2H2 | 203.9921686 | 16.27866998 | 0.616208626 | 0.089063065 | 0.009649567 | 0.034599807 | 1.061959723 | 1967.984272 | 23.44819479 |
| GTSE1 | 5.678858361 | 0.150746453 | 3.785476747 | 1.050705042 | 2.199873634 | 58.20243252 | 0.017634183 | 16690.64945 | 79744.22915 |
| HUS1 | 0.004069496 | 24.79458614 | 0.757787349 | 0.829370268 | 3.938051128 | 0.128681606 | 49.07183094 | 0.603728055 | 3225.625163 |
| IGHMBP2 | 0.912936575 | 6.454676586 | 0.394122329 | 0.012545828 | 1.505577291 | 0.067190924 | 7.999270249 | 33.56761978 | 11360.08028 |
| IP6K3 | 0.066238493 | 4.486692389 | 0.783086455 | 0.093462665 | 1.108693766 | 0.303669685 | 0.620306965 | 2152.302003 | 2924.084221 |
| XRCC6BP1 | 4.201106356 | 0.531260031 | 1.097155552 | 0.888375061 | 0.959390036 | 0.538791699 | 0.209128997 | 180.8530844 | 135.5045222 |
| LIG1 | 0.111493574 | 1.101635903 | 1.014941544 | 1.65594794 | 0.78008159 | 0.123710242 | 2.768914099 | 16.799172 | 12.03842553 |
| MAP2K6 | 0.939740667 | 2.579564318 | 0.592328205 | 0.34674952 | 1.044878663 | 0.682767695 | 7.819732287 | 143.0378875 | 12272.44946 |
| MAPK12 | 26.47687095 | 0.126688602 | 2.067618216 | 2.695476039 | 1.910770502 | 14.38052599 | 0.013134369 | 8445.405823 | 1785.016349 |
| MBD4 | 0.046524973 | 0.683371396 | 0.914641803 | 1.202461128 | 0.160384195 | 0.033386473 | 321.581058 | 0.19328139 | 7.895608692 |
| MLH1 | 0.058806828 | 0.582256811 | 2.911655359 | 1.553114573 | 0.012310169 | 0.53249904 | 8.176744526 | 20.18263943 | 5856.617564 |
| MLH3 | 7.175425115 | 5.952362899 | 0.656608146 | 0.652618449 | 0.446385148 | 1.335344081 | 1.635941495 | 14.14194796 | 78.7023125 |
| MNAT1 | 0.191427889 | 0.731027928 | 1.696103486 | 0.307725316 | 1.79337135 | 0.002406826 | 116.2340892 | 5.41489558 | 7.584387945 |
| MPG | 0.070925227 | 0.630741254 | 0.886555283 | 0.842382995 | 0.235547071 | 0.030793696 | 0.078683597 | 0.252162294 | 4.428675777 |
| MRE11A | 0.438874717 | 1.132719384 | 0.499173727 | 0.612018815 | 0.268080563 | 0.54801961 | 0.553083727 | 428.636914 | 5800.734536 |
| MSH2 | 0.003637951 | 1.32791123 | 0.627730213 | 1.235668004 | 0.657257794 | 0.003072144 | 154.1311775 | 2.852886916 | 4.355018226 |
| MSH3 | 0.116480931 | 0.776396117 | 0.422346864 | 0.853874342 | 7.188808716 | 0.178550291 | 40.47167025 | 5.832209675 | 2940.210478 |
| MUTYH | 1.01779088 | 0.61392486 | 1.600205277 | 0.46453122 | 1.323412254 | 1.530369639 | 0.284168257 | 168.8072275 | 2009.079098 |
| N4BP2 | 2.128013205 | 0.630921399 | 0.85099547 | 1.410220869 | 0.450563991 | 1.360997108 | 0.146806812 | 645.5900635 | 11974.05837 |
| NBN | 3.868631287 | 1.096725557 | 0.917098611 | 1.016692796 | 4.7357447 | 5.071950765 | 0.058487034 | 1088.479504 | 6911.895289 |
| NTHL1 | 0.005551607 | 0.930241731 | 0.633518772 | 0.806766308 | 0.568687143 | 0.007271617 | 76.42465273 | 0.460010839 | 7.782911662 |
| OGG1 | 0.507078104 | 1.227122693 | 0.749270749 | 0.574149432 | 0.983964962 | 1.095320729 | 17.66374407 | 11.05964872 | 44.8114646 |
| PCBP4 | 0.113317553 | 1.027954101 | 1.202225075 | 0.901634452 | 1.764850216 | 0.283427422 | 0.238822789 | 5.572684874 | 17.05315332 |
| PCNA | 6.308071033 | 0.529144168 | 0.28971678 | 1.170312958 | 4.256006715 | 0.365923011 | 36.88483955 | 4.925232669 | 11360.08028 |
| AIFM1 | 0.456524415 | 1.08086221 | 1.263848791 | 0.750133059 | 0.686153852 | 0.26569429 | 3.651063221 | 137.7034664 | 235.2473387 |
| PMS1 | 17.06963077 | 0.413849357 | 2.16083292 | 0.550157487 | 0.528891335 | 4.132066739 | 0.122909224 | 848.5619119 | 4659.54127 |
| PMS2 | 0.556536298 | 0.729721459 | 1.525949141 | 0.461850568 | 1.026533537 | 0.221174866 | 29.9443716 | 12.62417579 | 983.9635164 |
| PMS2P3 | 0.090141698 | 0.593016559 | 0.176235315 | 0.91883773 | 1.175914953 | 0.082966586 | 38.82377974 | 286.1718573 | 10898.02741 |
| PNKP | 0.084310223 | 1.124730618 | 0.728071683 | 0.909544069 | 0.995299214 | 0.107978392 | 58.06474351 | 3.621397149 | 12876.25797 |
| PPP1R15A | 47.92116435 | 0.697061897 | 0.663491422 | 1.336016877 | 12.07350696 | 0.988582155 | 0.608377664 | 136.6631537 | 81.52791322 |
| PRKDC | 0.017404824 | 1.818927013 | 1.907221994 | 0.42647035 | 0.21938645 | 0.00715759 | 106.4413111 | 2041.827854 | 275452.4096 |
| RAD1 | 12.54399364 | 0.481129481 | 0.935882861 | 1.911064825 | 1.857993458 | 16.90188271 | 0.023814335 | 3162.94402 | 25857.28595 |
| RAD17 | 2.976964564 | 0.913679767 | 1.022983949 | 0.954388562 | 0.258552071 | 0.126775644 | 194.2322286 | 0.385380456 | 22.73431607 |
| RAD18 | 0.112353578 | 1.339375318 | 1.578399632 | 1.372754092 | 1.481919146 | 0.079854712 | 15.41008259 | 0.65769154 | 2077.196496 |
| RAD21 | 6.308071033 | 0.014957023 | 410.5417233 | 0.113175992 | 1.108693766 | 0.141689357 | 10.32163409 | 900.4704613 | 11360.08028 |
| RAD50 | 1.223240084 | 25.94610132 | 0.926416183 | 2.8718232 | 1.696500433 | 0.00929927 | 42.63034491 | 1.719162426 | 11360.08028 |
| RAD51 | 6.308071033 | 8.885729427 | 1.490698783 | 0.608444427 | 0.220702545 | 0.010294734 | 18.30427799 | 256.5695684 | 468.8752206 |
| RAD51B | 1.55385703 | 2.402230742 | 45.75682097 | 1.131399325 | 22.74264322 | 1.15500347 | 0.046069495 | 1716.647959 | 5854.775049 |
| RAD9A | 0.009582765 | 16.03098764 | 0.802980772 | 0.800818027 | 0.000697094 | 0.09685955 | 43.286933 | 3.823446218 | 71276.6264 |
| RBBP8 | 0.285021604 | 0.863807152 | 1.144512061 | 1.850412538 | 0.834861488 | 0.078340439 | 0.197778009 | 0.265270297 | 11360.08028 |
| REV1 | 0.118231496 | 0.623464535 | 0.495047088 | 2.637407198 | 0.002307947 | 0.262534957 | 77.01303331 | 0.902190108 | 5.478493467 |
| RPA1 | 1.647663681 | 0.783428696 | 1.42859874 | 1.183641944 | 0.891835981 | 0.174400075 | 35.05357673 | 2.204138561 | 22.3395362 |
| SEMA4A | 0.004513006 | 0.952194974 | 2.807311487 | 1.266753644 | 1.704508476 | 0.00701405 | 278.554843 | 0.25766383 | 11360.08028 |
| SESN1 | 0.657191323 | 0.777700806 | 1.074092987 | 0.555501649 | 0.593111824 | 0.140857096 | 20.16246389 | 7.910508139 | 2341.461232 |
| SMC1A | 0.984618868 | 0.928002418 | 1.317769455 | 1.061300755 | 0.859823306 | 0.936011549 | 0.548967016 | 11.92598443 | 194.9935562 |
| SUMO1 | 0.141668097 | 0.953967854 | 1.190358536 | 0.673675711 | 0.726504393 | 0.207435104 | 31.10963473 | 0.508687252 | 4167.959384 |
| TP53 | 1.003559392 | 0.741687776 | 1.860308832 | 0.720797113 | 1.789525028 | 0.445477235 | 517.9148249 | 0.2149689 | 174.9603093 |
| TP73 | 0.007638542 | 0.805194599 | 0.439819467 | 0.755009812 | 0.482133958 | 0.002737386 | 1.700585174 | 1.067322414 | 63833.01415 |
| TREX1 | 57.08463426 | 0.231059197 | 0.012661552 | 2.430880346 | 16.26476222 | 47.45509162 | 0.014362725 | 3252.853212 | 9567.843276 |
| UNG | 2.42614621 | 1.109785572 | 0.473848178 | 2.505510725 | 3.526761746 | 0.149852176 | 0.046617113 | 3.964541443 | 8.3179646 |
| XPA | 0.387858708 | 1.266954629 | 1.472618715 | 2.694663656 | 0.111503376 | 0.235635839 | 0.366906397 | 315.139887 | 247.2513904 |
| XPC | 18.38434758 | 1.082881097 | 1.410658142 | 0.504132599 | 1.608222218 | 3.910501998 | 0.041694374 | 816.686912 | 996.8292705 |
| XRCC1 | 0.5460788 | 1.319962951 | 0.758654445 | 0.367624285 | 0.785485803 | 0.433250682 | 0.833787129 | 154.2853067 | 19792.90212 |
| XRCC2 | 27.22614926 | 0.244680375 | 0.652659865 | 0.988468455 | 1.108693766 | 67.55450336 | 0.025381961 | 570.2500354 | 4082.565278 |
| XRCC3 | 15.22637042 | 0.267308151 | 0.474280634 | 2.250271784 | 4.183251645 | 85.55845353 | 0.004746414 | 9497.418941 | 20654.02097 |
| ZAK | 4.064392746 | 0.881128185 | 1.020735929 | 0.566163174 | 1.540717024 | 0.614912218 | 0.293620999 | 266.5157271 | 4116.101696 |
